# Models for depression recognition and efficacy assessment based on clinical and sequencing data

**DOI:** 10.1016/j.heliyon.2024.e33973

**Published:** 2024-07-04

**Authors:** Yunyun Hu, Jiang Chen, Jian Li, Zhi Xu

**Affiliations:** a Key Laboratory of DGHD, MOE, School of Life Science and Technology, Southeast University, 210096, Nanjing, China; b Department of Psychosomatics and Psychiatry, Zhongda Hospital, School of Medicine, Jiangsu Provincial Key Laboratory of Brain Science and Medicine, Southeast University, Nanjing, 210009, China; c Research and Education Centre of General Practice, Zhongda Hospital, Southeast University, Nanjing, 210009, China

**Keywords:** Depression recognition, Depression efficacy, Deep learning, DNA methylation

## Abstract

Major depression is a complex psychiatric disorder that includes genetic, neurological, and cognitive factors. Early detection and intervention can prevent progression, and help select the best treatment. Traditional clinical diagnosis tends to be subjective and misdiagnosed. Based on this, this study leverages clinical scale assessments and sequencing data to construct disease prediction models. Firstly, data undergoes preprocessing involving normalization and other requisite procedures. Feature engineering is then applied to curate subsets of features, culminating in the construction of a model through the implementation of machine learning and deep learning algorithms. In this study, 18 features with significant differences between patients and healthy controls were selected. The depression recognition model was constructed by deep learning with an accuracy of 87.26 % and an AUC of 91.56 %, which can effectively distinguish patients with depression from healthy controls. In addition, 33 features selected by recursive feature elimination method were used to construct a prognostic effect model of patients after 2 weeks of treatment, with an accuracy of 75.94 % and an AUC of 83.33 %. The results show that the deep learning algorithm based on clinical and sequencing data has good accuracy and provides an objective and accurate method for the diagnosis and pharmacodynamic prediction of depression. Furthermore, the selected differential features can serve as candidate biomarkers to provide valuable clues for diagnosis and efficacy prediction.

## Highlights

1. Integrated DNA methylation sequencing data, SNP data, and clinical information for the first time, and established a predictive model of Major depressive disorder diagnosis for Major depressive disorder individuals through deep learning.
2. By integrating multi-dimensional clinical data and deep learning, a model of antidepressant treatment effect in individuals with major depression was established.
3. The results of this study show that the machine learning and deep learning algorithm based on clinical and sequencing data has good accuracy and provides an objective and accurate method for the diagnosis and pharmacodynamic prediction of depression.

## 1 Introduction

Major depressive disorder (MDD) is a multifaceted mental illness involving genetic, neurological, and cognitive factors. It is characterized by negative tendencies in emotion and suicidal tendencies in behavior. At present, MDD affects more than 350 million people worldwide [1]. Among them, more than 98 million people in China suffer from MDD, and the suicide rate accounts for 50 %, higher than the global average of 3 % [2,3] According to the World Health Organization (WHO), MDD will have the highest global burden by 2030 [4].

However, the etiology of depression is unknown, but it is thought that genetic predisposition and environmental factors are influential. Such as stressful life experiences will exert epigenetic modification in these risk genes via DNA methylation to generate long-lasting effects on these gene expression, which in turn cause brain structural and functional alteration and finally increase the vulnerability to depressive disorders. Preclinical and clinical studies have shown significant correlations between stress and depression [5], stress and epigenetic [6], and depression and epigenetic changes [7]. DNA methylation is one of the most important epigenetic modifications and is closely related to depression [[8], [9], [10]]. DNA methylation of BDNF gene can be used as a biomarker for differentiating healthy controls (HCs) from patients with depression due to different methylation levels of CpG units between HCs and patients with depression (DPs) [11]. DNA methylation in the 5-hydroxyptamine receptor (5-HTR) family, which includes HTR1A/2A/3A, is also associated with MDD [12,13]. Selective serotonin reuptake inhibitors (SSRIs) were the most commonly used antidepressants [14]. However, it does not work for all depressed people. Studies have suggested that functional genetic variants in HTR1A and HTR1B may be involved in individual differences in SSRI treatment response [15,16]. Thus, epigenetic biomarkers such as DNA methylation, which is found in depressions and is thus important in depression development can help diagnose and predict response to adjuvant chemotherapy [17].

Methylation is a chemical modification of DNA molecules that involves the addition of methyl groups in DNA molecules [18]. Sequencing data typically contains information about the DNA sequence and can therefore be used to detect methylation patterns in DNA [19]. DNA methylation, which does not involve changes in DNA nucleotide sequence, occurs mainly at CpG site, the region adjacent to cytosine nucleotide and guanine nucleotide, and is one of the most studied epigenetic mechanisms [[20], [21], [22]]. DNA methylation plays a key role in determining genome structure and function, including regulating cell differentiation and coordinating gene expression [23,24]. DNA methylation disorders are associated with the development of atherosclerosis, cancer, obesity, type 2 diabetes, neuropsychiatric disorders, and other complex multifactorial diseases, and predict all-cause mortality [20,[25], [26], [27], [28], [29]]. Single nucleotide polymorphisms (SNPs) are single base variations in a DNA sequence and are one of the most common forms in the genome [30]. While most SNPs have no biological consequences, a small number of alternatives have functional significance and are fundamental to human diversity. Moreover, there are complex interactions between methylation variants and SNPs, for example, variations at SNP sites may affect nearby or distant methylation patterns, thereby affecting gene expression or regulation [31].

Early detection and intervention can prevent progression, and predicting a patient’s response to treatment can help select the best treatment. However, at present, the clinical diagnostic methods of MDD are mainly based on the diagnostic criteria of MDD in the International Classification of Diseases 10 (ICD-10) or the Diagnosis and Statistics of Mental Disorders V (DSM-V) [32], combined with doctor-patient communication, scale analysis and doctors' diagnostic experience [33,34]. This method is easily affected by subjective factors such as the degree of patient cooperation and the doctor’s proficiency, which may lead to misdiagnosis or missed diagnosis.

In recent years, with the rapid development of medical big data and artificial intelligence technology, machine learning (ML) and deep learning (DL) have been gradually applied in the early prediction and auxiliary diagnosis of depression due to their powerful data processing capabilities. ML and DL can capture features from large, multidimensional, and interconnected datasets and train them using automatic learning algorithms to find hidden phenotypic or genotypic structures and then predict treatment responses based on individual patient characteristics [35]. A recent study using ML to predict antidepressant treatment response in MDD patients identified 25 characteristics that best predicted whether patients would respond to citalopram antidepressants and predicted the persistence, chronic, and severity of depression using a self-reported questionnaire [36,37]. A study by Kautzky et al. demonstrated that a random forest prediction model for treatment outcome correctly identified 25 % of responders by using 3 SNPs (including BDNF rs6265, PPP3CC rs7430, and HTR2A rs6313) and a clinical variable [38]. In addition, Patel et al. employed an alternating decision tree approach to predict treatment outcomes with 89 % accuracy in patients with late-life depression by using structural imaging data and clinical variables [39]. There are ways to improve the accuracy of models by screening for some of the clinical features of patients with depression [40,41].

Nevertheless, the current methodologies suffer from inherent limitations, characterized by either a lack of precision or undue complexity [38]. Consequently, the imperative exists to devise more streamlined and precise diagnostic techniques for depression or to unearth valuable biomarkers for forecasting depression treatment efficacy. This investigation delves into substantial disparities between individuals afflicted with MDD and HCs, exploiting these pronounced distinctions as a potential avenue to identify diagnostic and prognostic markers for depression. This pursuit is based on the integration of both sequencing and clinical diagnostic data, factoring in multiple variables for optimizing predictive accuracy.

## 2 Materials and method

### 2.1 Study design

**Fig. 1:**
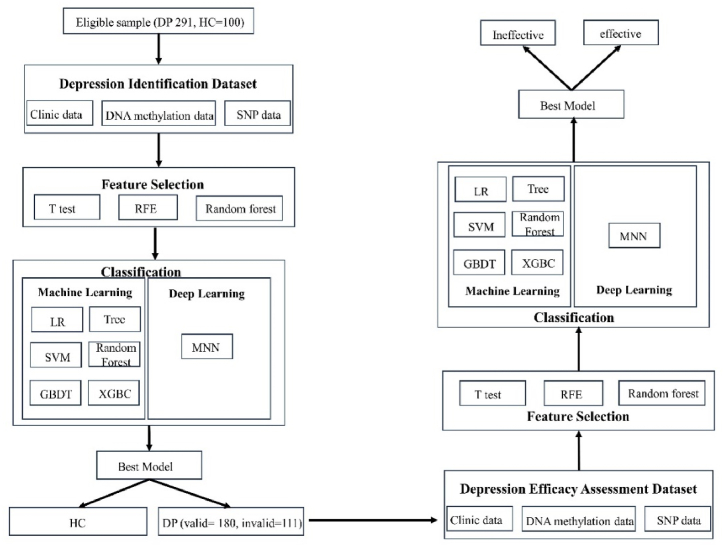
Experimental design of this study.

The analysis of depression identification and prognosis assessment are summarized in Fig. 1.

Multiple feature selection methods were carried out to find the best feature subset for the detection of depression patients and the efficacy of antidepressants. Seven binary classifiers were utilized to calculate the classification performances. RFE: recursive feature elimination; LR: logistic regression; SVM: support vector machine; GBDT: gradient boosted decision tree; XGBC: gradient boosting classifier; MNN: multiple neural network; MI: mutual information.

### 2.2 Subjects and clinical evaluation

**Fig. 2:**
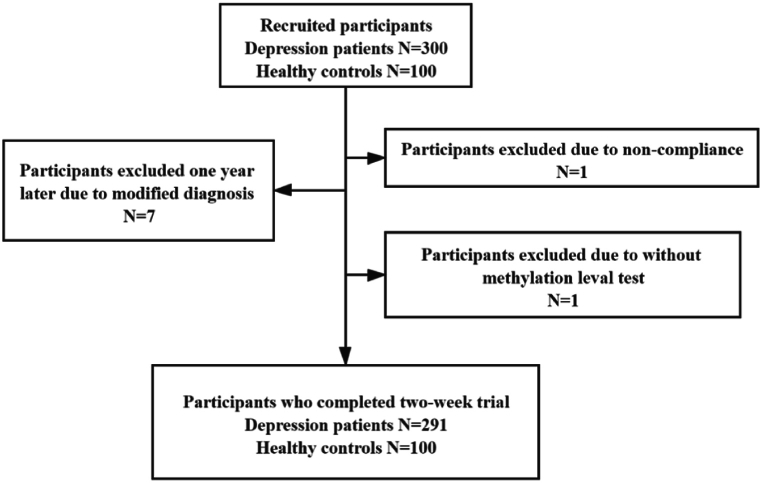
Participants recruitment flow chart during the experiment.

A total of 300 patients with MDD and 100 HCs were recruited for the study, which was referred by Zhongda Hospital. As shown in Fig. 2, 291 DPs, and 100 HCs were retained for subsequent analysis.

According to the criteria from the Diagnostic and Statistical Manual of the American Psychiatric Association (APA), fourth edition (DSM-IV), all recruited DPs met the criteria for diagnosis of depression.

#### 2.2.1 Antidepressant treatment and outcome assessment

The MDD patients were treated with a single antidepressant as recommended by a clinician (SSRI: n = 174, non-SSRI: n = 112). Before the study began, the assessment and treatment protocols used by psychiatrists were standardized. Other than supportive medical visits, the MDD patients did not receive other psychological interventions. The study excluded patients who needed to change antidepressants or performance noncompliance. The severity of depression symptoms was assessed with the HAMD-17 score at baseline and 2 weeks later. The effective, outcome response was defined as a reduction ratio of ≥50 % from the baseline HAMD-17 score after 2 weeks of treatment, based on the guideline of the World Federation of Societies of Biological Psychiatry. Otherwise, it was defined as ineffective.

#### 2.2.2 Gene polymorphism and methylation detection

**Fig. 3:**
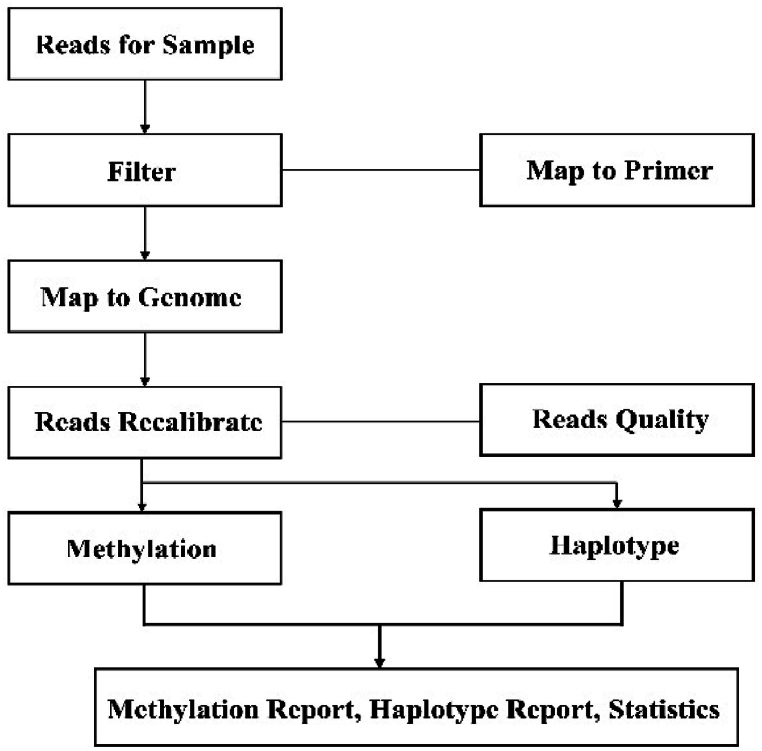
Sequencing flow chart.

To fully detect biochemical markers, peripheral blood samples were collected from subjects at 8:00 a.m. at least 8 h after eating (collected before taking the medication). Blood samples for methylation analysis were collected in a 5 ml EDTA tube and stored at −80 °C until genotyping. DNA methylation level was analyzed by MethyTarget® (Genesky Biotechnologies Inc. Shanghai, China), a multi-target CpG methylation analysis method based on NGS. According to the manufacturer’s protocol, sequencing was performed on the Illumina HiSeq platform in a 2x150 bp paired-end mode. The data obtained by sequencing was analyzed (Fig. 3). The average effective sequencing depth of target regions for all samples was 600 X, more than 80 % samples have an effective depth greater than 400-fold, and 90 % samples exceed 10-fold. After rigorous quality control, 406 eligible CpG sites were detected in 300 depressed patients and 100 healthy controls. A total of 23 SNPs of the four candidate genes met the DNA methylation status criteria of the sequence to be tested for subsequent analysis. There were 5 SNP sites in HTR1A, 3 SNP sites in HTR1B, 1 SNP site in S100A10 and 14 SNP sites in BDNF.

#### 2.2.3 Statistical analysis

Clinical and demographic data were given as mean ± standard deviation. Differences with a *P*-value <0.05 were considered statically significant. T-test was used to identify differences in continuous variables between groups. Differences in categorical variables were compared using the standard chi-square test. The statistical analysis was implemented using the scipy package of Python. Scipy (version number: 1.4.1) package of Python was used for differential statistical analysis. The Hard Weinberg equilibrium (HWE), the percentage of undeleted loci, and the minor allele frequency of SNP data were analyzed using Haploview 4.2.

### 2.3 Data preprocessing

The collected data contains noise, missing values, unbalanced and non-numerical data. Before feature selection and modeling, we need to preprocess the data, including missing value processing, feature coding, data normalization, and data integration. After pretreatment, the overall quality of big data can be greatly improved.

#### 2.3.1 Data cleaning

These different missing data types were disposed of in different filling methods. Over 20 % of data loss is directly discarded, and less than 20 % loss data can be filled. The missing values of DNA methylation data and SNP data were deleted in this study. As for the clinical indicators, we adopt the mode value to fill the discrete indicators. However, in order to avoid the disadvantage of over-concentration of statistical filling values (mean, median and mode). Based on this, this study intends to use random filling method to interpolate continuous features. That is, substitute values are randomly extracted from the observation data of known features to fill the missing values, which can effectively avoid the fixity of data filling.

#### 2.3.2 Feature coding

The value of DNA methylation level is a set of quantitative data between 0 and 1. For the encoding of methylation data, the median of each site was taken as the threshold. It was coded as 1 if it was greater than the median, or as 0. In addition, One-hot coding was applied to the upper-class genes of chromosomes, sex, education level, insomnia, suicide, and marital status.

#### 2.3.3 Data normalization

The data normalization step is used to scale the feature values for several reasons:

(1) To avoid features in greater numeric ranges dominating those in smaller numeric ranges;
(2) To improve the efficiency and convergence rate;
(3) To get higher classification accuracy. Here we used maximum-minimum normalization to normalize the data (also called features) into a fixed range between the maximum and minimum. Each feature can be scaled to range [0, 1] as follows:

[1] $$X=\frac{x-x_{\min}}{x_{\max}-x_{\min}}$$

where x is the original value, x_min_ and x_max_ are the lower and upper bounds of the feature values, and the X is the scaled value.

#### 2.3.4 Data integration

**Fig. 4:**
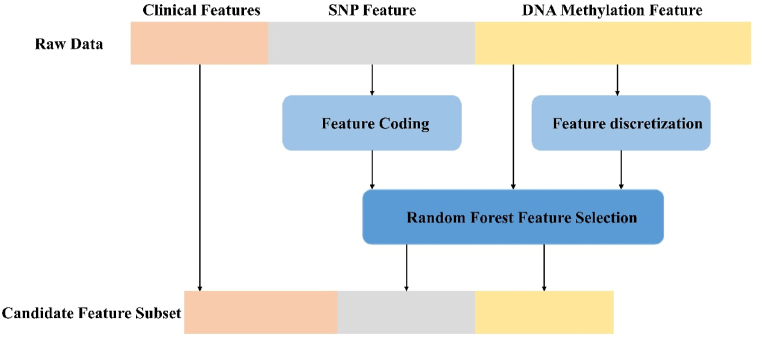
Data integration diagram.

Data integration involves combining heterogeneous data from different sources to better understand for subsequent analysis. Fig. 4 shows the data integration.

### 2.4 Feature engineering

Feature engineering is the process of converting raw data into features that better express the nature of a problem. Simply put, feature engineering is to find the features that have a significant impact on the dependent variable, so that these features can be applied to the prediction model to improve the prediction accuracy of invisible data. The depression recognition data set and depression efficacy assessment data set have the characteristics of a large feature number and small sample number. The feature selection method in feature engineering is used to identify the relevant feature subset in high-dimensional data and construct feature engineering based on it. This part is implemented by Python 3.7.6. T-test is conducted in the scipy 1.4.1 of Python. Other algorithms are carried out by the Python package sklearn 0.22.1.

#### 2.4.1 Filter method

The filter method is based on the feature inherent characteristic to select feature subsets and does not involve any learning algorithm, which includes T-test and Chi-square test. Where T-test was performed to analyze the degree of difference between two types of samples for a certain feature. The significance level of the T-test was determined as 0.05, the T-value of each feature was calculated and the final difference feature was determined. The larger the statistic value is, the smaller the p-value is, which indicates the greater the importance of the feature. Chi-square is a correlation filtering specifically for classification problems. It calculates the chi-square statistics between each non-negative feature and the label, and ranks the features according to the chi-square statistics from high to low. T-test was used to screen the differential features for continuous features in DNA methylation data and clinical data, and the chi-square test was used to select the differential features for discrete features.

#### 2.4.2 Wrapper method

In wrapper method, predictors are regarded as black boxes, while the performance of predictors is treated as an objective function to evaluate feature subsets. The recursive elimination method (RFE), a popular wrapper method, drops the feature with the lowest feature coefficient returned by an iterative train classifier. If multiple feature subsets reach the best accuracy, the subset with the least features is returned. Through continuous iteration, the feature subset is optimized to improve the discriminant performance.

#### 2.4.3 Embedded method

Embedded method tunes feature selection and classifier as one. That is feature selection is a part of the training process. In this study, the feature importance of random forest was used for feature selection in embedded method. Random forest is a practical ensemble learning method, which is based on decision tree model and bagging to integrate multiple decision trees to form a forest. Then, the last prediction outcome is gained by voting through the multiple decision trees. This study used the Gini coefficient, which is widely used at present, as the branch rule. Specifically, the training set is used to build multiple classification trees, while the test set examines the importance score of each predictor in the decision tree one by one. The average value of the importance score of the predictor in all decision trees is regarded as the importance index of the predictor. The importance of predictors is ranked from high to low. The final feature subset is selected by the out-of-bag prediction error rate.

### 2.5 Classification algorithms

Classifiers may reach drastically different performances on the same dataset. In this study, seven popular classifiers were utilized to evaluate the degree of differentiation of feature subsets between two types of samples, including six machine learning algorithms (logistic regression, decision tree, SVM, random forest, GBDT and xgboost) and a backpropagation deep neural network model.

This study uses default Settings to implement logistic regression (LR), decision tree (DT) and SVM classifiers. Ensemble learning accomplishes learning tasks by building and combining multiple weak classifiers. Random forest algorithm integrates multiple decision trees through the idea of ensemble learning. Its basic decision unit is decision tree. The random forest (RF) algorithm integrates all decision tree classification results, and formulates the category with the most votes as the final output. Gradient boosting decision tree (GBDT) is an iterative algorithm that achieves the purpose of data classification by using an additive model. That is, the linear combination of basic functions reduces the residuals until clearly classification in the training process. XGBClassifier (XGBC) ensemble the individual prediction results and receives the most votes as the final results based on a series of weak classifiers. The six ML models were built on sklearn (version: 0.19.0) package of python.

Deep neural network is a feedforward neural network in deep learning. The deep learning model used in this study is based on the deep learning frame framework of Keras. The model consists of three layers: For the input layer, hidden layer, and output layer, relu is selected for the activation function of the input layer and hidden layer, and sigmoid is selected for the activation function of the output layer. dropout is used to prevent overfitting of the model, and random gradient descent is used to optimize the model parameters. The number of neurons, learning rate, number of training rounds, and batch data size should be adjusted according to the actual situation. At least 200 evaluations were conducted for each model evaluation.

### 2.6 Performance measurements

The area under the receiver operating characteristic curve (ROC) was calculated to evaluate the performance of the prediction model. AUC is a more objective performance evaluation index, which is popular with most researchers to evaluate the performance of the model. The better the prediction model, the higher the AUC. In addition, to better describe the overall performance of the prediction model, measures, such as accuracy (ACC), precision, recall, and F1 score were also used to evaluate the performance of classifiers, respectively. The precision and recall are integrated into the F1 score. The closer it is to 1, the better the model. In addition, ten-fold cross-validation was utilized to calculate the binary classification performance. Specifically, the whole dataset will be divided into 10 separate small datasets. At this point, a 9/10 dataset is used to train the predictive model. The remaining 1/10 dataset is used as a test set to test the performance of the predictive model.

## 3 Result

### 3.1 Analysis of the dataset for identifying DPs and HCs

#### 3.1.1 Demographic and clinic characteristics of the participants

The demographics and clinical characteristics of the DPs and HCs were shown in Table 1. There were no significant differences in sex and age between DPs and HCs. NLES (Negative Life Events Scale score) [42], CTQ (Childhood Trauma Questionnaire score) [43], and one-NLES scores showed significant differences between DPs and HCs, as well as the NLES, CTQ and one-NLES of DPs were significantly higher than HCs. These scale data constitute clinical data.

**Table 1:** Demographic and clinical characteristics of the participants.

Demographic and clinical indexes		DP (Depression Patient)		HC (Healthy Control)		*P*-value
		N = 291	%	N = 100	%	
Sex	Female	189	64.95	64	64.00	0.9602
	Male	102	35.05	36	36.00	
CTQ (mean ± SD)		47.93 ± 8.88		43.70 ± 3.12		7.9666e-10
Age (mean ± SD)		46.41 ± 13.64		46.33 ± 14.27		0.9608
NLES (mean ± SD)		25.45 ± 34.14		7.56 ± 16.79		1.3780e-08
one-NLES (mean ± SD)		4.58 ± 9.84		0.26 ± 1.50		2.0759e-10
Life	0	24	8.25	39	39.00	1.6823e-12
	1	267	91.75	61	61.00	

CTQ: the Childhood Trauma Questionnaire score; NLES: the negative life events scale score; one-NLES: the negative life events scale score in one year; life: whether or not a negative event happened, where 0 means it has not happened and 1 means happened; N: sample size.

#### 3.1.2 Sequencing characteristics of participants

**Fig. 5:**
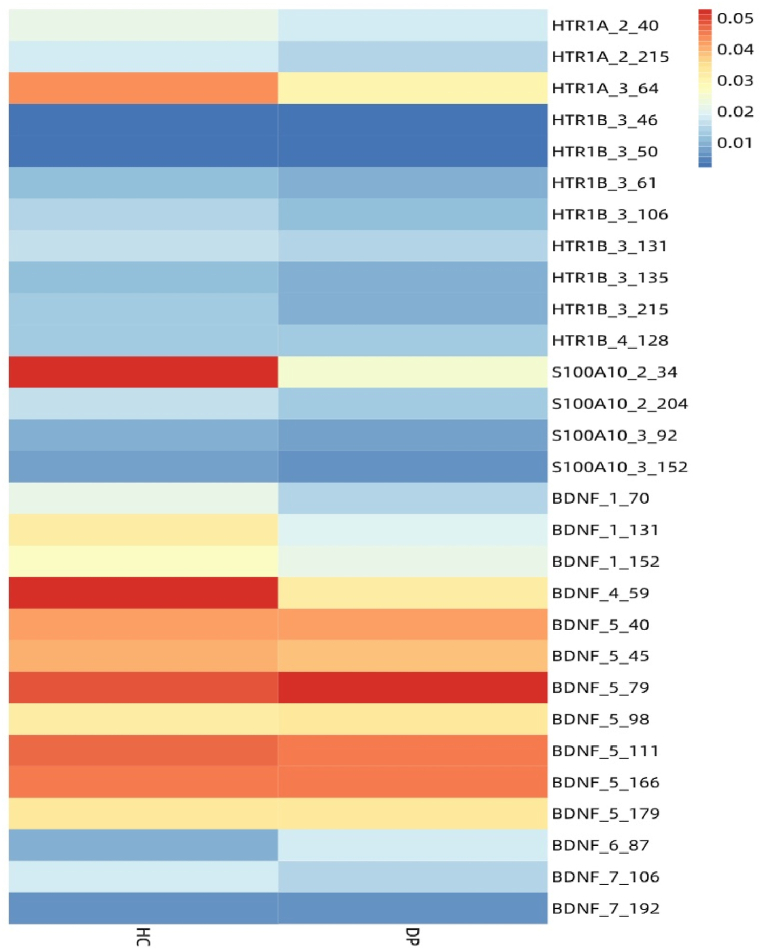
Visualization of differential CpG sites in DPs and HCs.

A total of 391 peripheral blood samples (291 DP samples and 100 HC samples) from recruiters were sequenced on the Illumina HiSeq sequencing platform. After strict quality control, a total of 406 CpG sites were obtained. Methylation levels of CpG sites were calculated with the ratio of methylated cytosines over total cytosines, which is defined as β. The β value is between 0 and 1, and 1 indicates complete methylation of the site. Statistical analysis of 406 CpG sites found that only 29 sites showed significant differences between DPs and HCs (Fig. 5). We also paid attention to the association among DPs, HCs and SNP data. A total of 23 SNP sites of 4 candidate genes were obtained. After the One-Hot coding process, the 23 SNPs became 68 features. After multiple comparative analysis, it was found that 22 SNP sites did not reach the significant level between DPs and HCs. The basic information of SNP data is shown in Table 2.

**Table 2:** General characteristics of SNP site.

Gene	Name	%gene	HWpval	MAF	Alleles
BDNF	rs2049048	100	0.8728	0.062	G:A
	rs2883187	98.5	0.5423	0.433	G:A
	rs7944119	94.6	0.0711	0.03	G:T
	rs4923468	93.8	0.348	0.023	C:A
	rs727155	99.7	1	0.053	C:T
	rs1491850	100	0.1043	0.481	T:C
	rs2049046	99	0.6781	0.435	T:A
	rs7931247	99.7	1	0.447	T:C
	rs16917271	90.5	0.6929	0.148	T:A
	rs6265	100	0.4054	0.491	T:C
	rs13306221	99.7	0.2927	0.021	C:T
	rs7119334	100	0.6457	0.285	A:C
	rs71311904				-:CATTT
	rs56164415	97.4	0.2641	0.02	G:A
HTR1A	rs6295	99.7	0.6499	0.256	G:C
	rs10042486	78.1	0.012	0.246	T:C
	rs6449693	100	0.2971	0.202	A:G
	rs878567	100	0.4366	0.204	G:A
	rs6294	99.5	1	0.051	C:T
HTR1B	rs6297	100	1	0.121	T:C
	rs6296	100	1	0.43	G:C
	rs6298	99.7	0.8112	0.428	A:G
S100A10	rs113674295				-: AGAAAAGCTCTGCTGTGTA

%gene: the percentage non-missing for this SNP site; HWpval: the Hardy Weinberg equilibrium p value; MAF: the minor allele frequency for this SNP site; alleles: the major and minor alleles for this SNP sites.

#### 3.1.3 Machine learning and deep learning predictive results

**Fig. 6:**
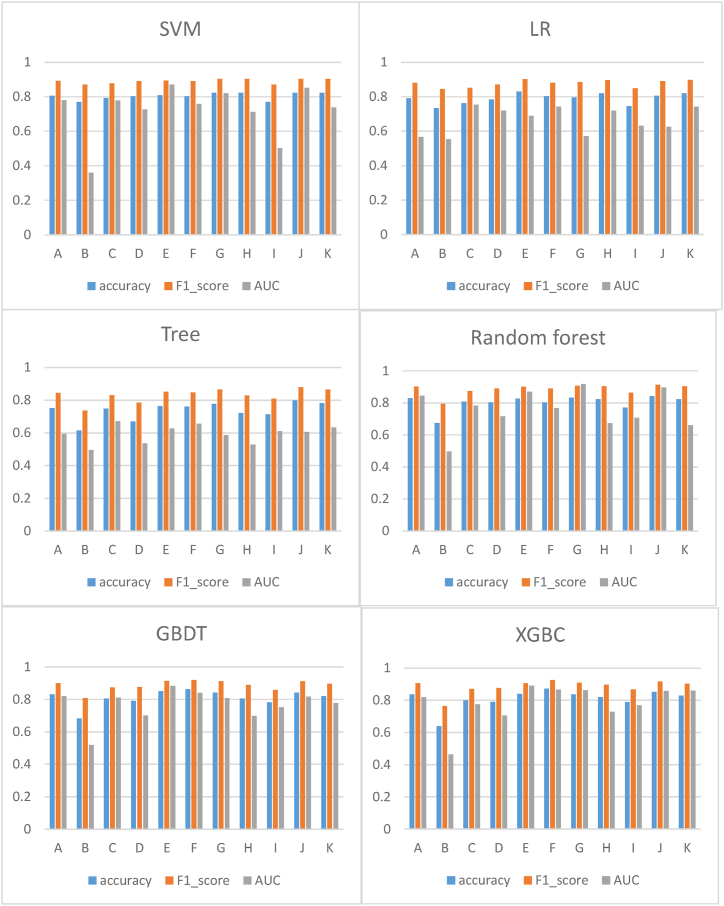
Prediction results of six ML algorithms on 11 different datasets.

Based on three independent datasets, we conducted independent analysis and combined analysis, respectively. Six ML algorithms (LR, DT, SVM, RF, GBDT and XGBC) were used for predictive analysis on 11 different datasets (methylation dataset; SNP dataset; clinic dataset; methylation encoding dataset; the dataset of methylation and clinic; the dataset of methylation encoding and clinic; the dataset of methylation and SNP; the dataset of methylation encoding and SNP; the dataset of SNP and clinic; the dataset of methylation combined SNP and clinic; the dataset of methylation encoding combined SNP and clinic). Fig. 6 shows that the ensemble learning prediction models (RF, GBDT and XGBC) were better than single prediction models (LR, DT and SVM) in general.

Since different classifiers produce different results for the same feature set, we compared the prediction results of six machine learning algorithms (LR, DT, SVM, RF, GBDT and XGBC) and a DL algorithm (backpropagation deep neural network model). To avoid the randomness of the dataset partition, we used ten-fold cross-validation to calculate the binary classification. Table 3 shows the prediction results of six machine learning algorithms and a DL algorithm. For the dataset of methylation, SNP and clinic, machine learning achieved good performance in distinguishing DPs from HCs. In general, the RF prediction algorithm has the best prediction effect on the dataset of methylation, SNP and clinic. While the DL algorithm has a poor effect among the seven algorithms, which is mainly reflected in the score of AUC, and F1 score (integration of precision and recall). This may be due to the fact that the amount of data in machine learning is within a certain range due to deep learning, which is also proven in industry.

**Table 3:** Prediction results of six ML algorithms and a DL algorithm on the dataset of methylation, SNP and clinic.

	LR	DT	SVM	RF	GBDT	XGBC	DL
Accuracy	0.8048	0.7989	0.8234	0.8422	0.8420	0.8513	0.82382
Precision	0.8232	0.8613	0.8234	0.8403	0.8523	0.8549	0.411
Recall	0.9716	0.9033	1	1	0.9833	0.9886	0.5
F1_score	0.8909	0.8797	0.903	0.9128	0.9113	0.9164	0.446
AUC	0.6251	0.6058	0.8519	0.8951	0.8165	0.8586	0.528

All predictive performance is mean value.

A: methylation dataset; B: SNP dataset; C: clinic dataset; D: methylation encoding dataset; E: the dataset of methylation and clinic; F: the dataset of methylation encoding and clinic; G: the dataset of methylation and SNP; H: the dataset of methylation encoding and SNP; I: the dataset of SNP and clinic; J: the dataset of methylation combined SNP and clinic; K: the dataset of methylation encoding combined SNP and clinic.

#### 3.1.4 Experimental results and analysis of feature selection

First, the features of 406 CpG sites, 7 clinical features, and 68 SNP sites were ranked by importance score from largest to smallest, and then a stepwise RF was performed to identify the optimal subset of features associated with the outcome variable. Based on the experimental analysis, we summarized the selected features in Table 4. Then, the model is modeled. Compared with the original data model, the predictive performance of the model constructed by using the feature subset after dimensionality reduction of RF is greatly improved. Table 5 shows the prediction results of the feature subset DL model after dimension reduction of RF. The accuracy of the model is 87.274 %, an increase of 6.07 %, the F1_score is 76.90 %, an increase of 72.42, and the AUC is 85.10 %, an increase of 61.17 %.

**Table 4:** Results of RF feature selection.

Dataset	Feature number before dimensionality reduction	Feature number after dimensionality reduction
Methylation	406	4
Methylation encoding	406	30
SNP	68	1
Methylation + clinic + SNP	481	18
Methylation encoding + clinic + SNP	481	60

**Table 5:** Prediction results of the DL model of feature subsets after RF dimensionality reduction.

dataset	Accuracy	Precision	Recall	F1_score	AUC
4methylation + clinic	0.83032	0.788	0.581	0.58	0.728
4methylation + clinic+1SNP	0.82273	0.41	0.50	0.451	0.662
30methylation encoding + clinic	**0.87274**	**0.844**	**0.752**	**0.769**	**0.851**
30methylation encoding + clinic+1SNP	0.82705	0.656	0.57	0.567	0.826
Clinic+1SNP	0.76276	0.385	0.495	0.431	0.645
18 Methylation + clinic + SNP	0.8275	0.462	0.517	0.477	0.57
60Methylation encoding + clinic + clinic + SNP	0.84154	0.667	0.594	0.592	0.848

Then we used *P*-value≤0.05 as the differential screening threshold. A total of 23 differential features were obtained from methylation, clinical and SNP datasets, including 20 methylation features, 2 clinical features and 1 SNP feature. The accuracy of DL prediction results of 23 feature subsets is 82.70 %, F1_score is 49.70 %, and AUC is 80.5 %. 33 differential features were obtained from the methylation, clinical, and SNP datasets, including 31 methylation coding features, 1 clinical feature, and 1 SNP feature. For the prediction performance of 33 feature subsets, the DL algorithm found that the accuracy of the model was 83.85 %, the accuracy of F1_score was 56.47 %, and the AUC was 81.35 %.

**Fig. 7:**
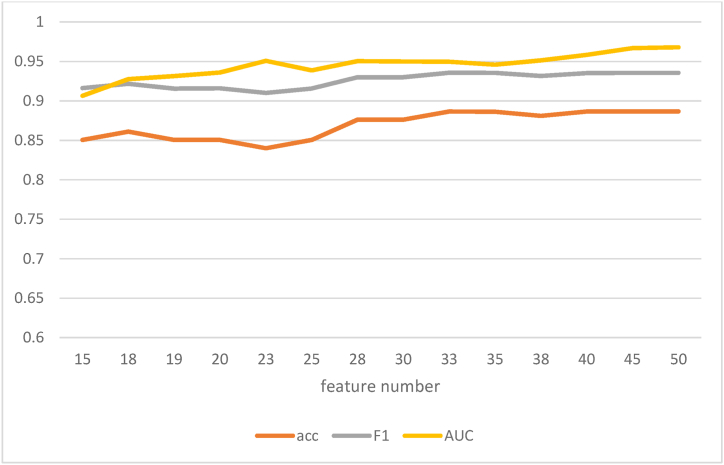
The change relationship between feature number and accuracy, F1 and AUC.

Finally, we then selected a RFE algorithm in the methylation combination of SNPs and clinical datasets for evaluation and feature selection. The score of each feature is constantly adjusted in repeated iterations. LR model was repeatedly constructed using RFE based on the variation maps of accuracy, F1_score and AUC in the process of screening optimal feature subsets of methylation, clinical and SNP data sets (Fig. 7). The two goals of reducing the number of features and improving the predictor are combined. We choose a feature subset of 18 features to build the model. The accuracy rate was 86.11 %, F1_score was 92.17 %, and AUC was 92.77 %.

In general, among the several methods, RFE was used to screen out 18 different features, which could effectively distinguish between depressed patients and healthy controls, with the highest accuracy of 87.26 % and AUC of 91.56 %, and the prediction effect was better than clinical judgment.

### 3.2 Analysis of patients after antidepressant treatment

#### 3.2.1 Demographic and clinic characteristics of the DPs

Table 6 describes the demographic and clinical features of the subjects. Among them, there were significant differences in gender, relapse times, HAMD-0W score of the subjects in the effective group and the ineffective group. The HAMD-0W score of the effective cohort was significantly lower than the ineffective cohort. Surprisingly, the females in effective group were more than the males.

**Table 6:** Demographic and clinic characteristics of the depression patient subjects.

Demographic and clinical indexes		Effective		Ineffective		*P*-value
		N = 180	%	N = 111	%	
Gender	Female	127	70.56	62	55.86	0.0153
	Male	53	29.44	49	44.14	
Education Level	Primary School or Below	29	16.11	20	18.02	0.6681
	Middle school	112	62.22	36	32.43	
	University and above	38	21.11	27	24.32	
Age		46.95 ± 13.20		45.50 ± 14.30		0.3821
HAMD-0W		22.50 ± 3.85		23.59 ± 4.04		0.0302
First_morbidity_age		41.93 ± 13.55		40.04 ± 14.75		0.2743
SHAPS score		6.59 ± 4.67		5.90 ± 4.42		0.2126
BSI-CV score		6.21 ± 7.64		6.99 ± 7.96		0.4109
TAS score		60.27 ± 9.16		60.92 ± 8.89		0.5576
SSRS score		33.46 ± 8.67		32.23 ± 8.12		0.2365
Diagnositc_decision	D	82	45.56	45	40.54	0.4738
	F	98	54.44	66	59.46	
Marital status	Spinsterhood	19	10.56	15	13.51	0.6088
	Married	150	83.33	86	77.48	
	Divorce	6	3.33	4	3.60	
	Widowed	2	1.11	3	2.70	
Relapse times	1	86	47.78	46	41.44	0.0455
	2	55	30.56	23	20.72	
	3	36	20.00	34	30.63	
Cause	F	37	20.56	20	18.02	0.7056
	T	143	79.44	91	81.98	
Suicide	F	81	45.00	41	36.94	0.2520
	T	95	52.78	66	59.46	
Mental_symptoms	F	166	92.22	97	87.39	0.5053
	T	10	5.56	9	8.11	
Insomnia	F	12	6.67	8	7.21	0.9764
	T	164	91.11	99	89.19	
medicine_classification	SSRI	108	60.00	66	59.46	0.9198
	Non SSRI	71	39.44	41	36.94	

Tips: A total of 291 DPs matched the inclusion criteria but did not meet the exclusion criteria, which completed 2 weeks of treatment. Among them, 180 DPs were effective in antidepressant treatment, and 111 were ineffective. The effective rate of treatment was 61.86 %. In the subsequent analysis, 18 clinical biomarkers were used, including age, gender, marital status, HAMD-0W, relapse times before enrollment, etc. HAMD-0W: HAMD-17 scores at week 0; SHAPS score: Snaith-Hamilton Pleasure Scale score; TESS score: Treatment Emergent Symptom Scale score; BSI-CV score: Beck Scale score for Suicide Ideation-Chinese Version; TAS score: Toronto Alexithymia Scale score; SSRS score: Social Support Rating Scale score; SSRI: selective serotonin reuptake inhibitors; T: true, it means the patients have this symptom; F: false, it means no this symptom.

#### 3.2.2 Sequencing characteristics of DPs

**Fig. 8:**
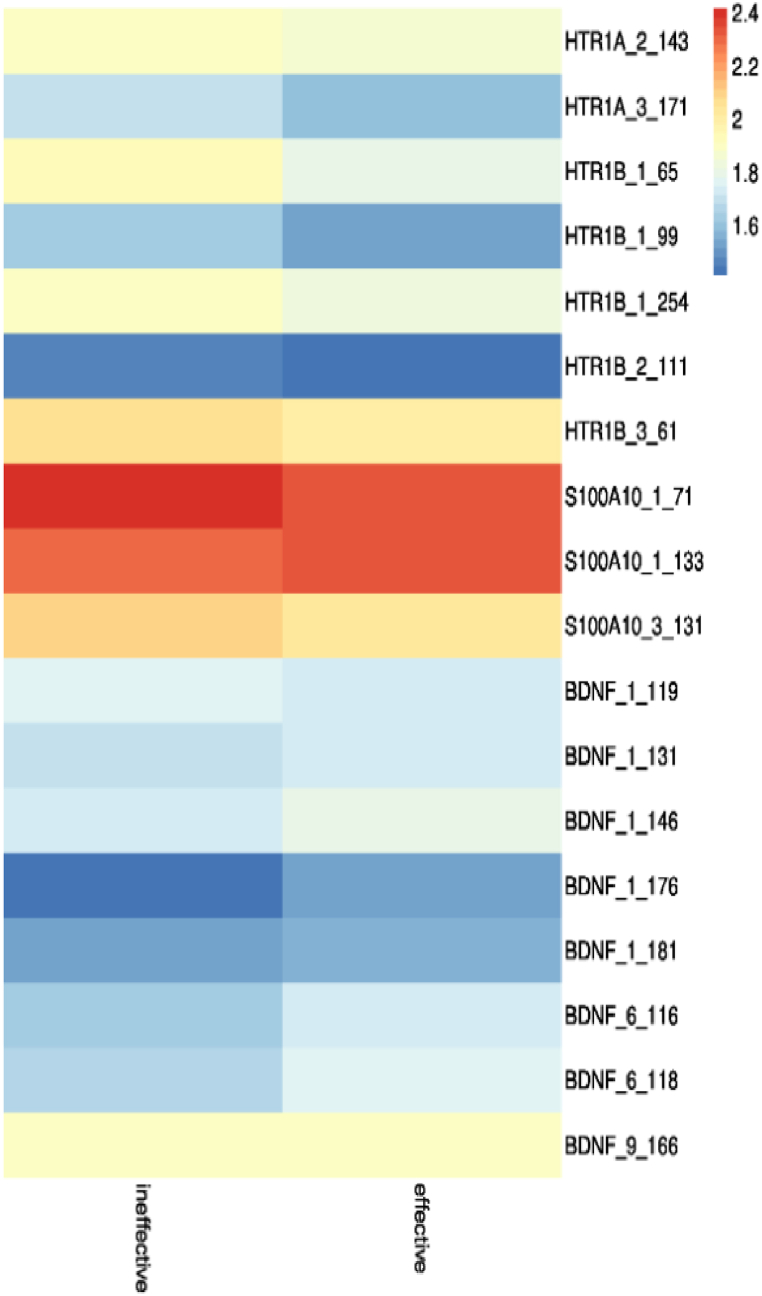
Heatmap of differential CpG sites for effective and ineffective groups.

The candidate genes' DNA methylation sequencing analysis was performed on the peripheral blood samples from 291 DPs. After strict quality control, there were a total of 406 CpG sites. Statistical analysis of 406 CpG sites found that only 18 CpG sites were significantly different between the effective group and the ineffective group. According to our previous studies, the primers were designed to cover 100 bp upstream and 100 bp downstream of four candidate genes (HTR1A, HTR1B, S100A10, BDNF) SNP sites. As shown in Fig. 8, there were 22 effective SNP sites captured. The basic information of SNP data is shown in Table 7. Analysis for single sites did not reveal any significant associations between the effective group and the ineffective group.

**Table 7:** General characteristics of SNP site.

Gene	Name	%gene	HWpval	MAF	Alleles
BDNF	rs2049048	100	1	0.062	G:A
	rs2883187	98.3	0.5398	0.419	G:A
	rs7944119	94.5	0.07	0.036	G:T
	rs4923468	93.1	0.3623	0.028	C:A
	rs727155	99.7	1	0.057	C:T
	rs1491850	100	0.1203	0.491	T:C
	rs2049046	98.6	0.5132	0.418	T:A
	rs7931247	99.7	0.8658	0.424	T:C
	rs16917271	91.7	0.4098	0.137	T:A
	rs6265	100	0.2872	0.481	T:C
	rs13306221	99.7	0.34	0.026	C:T
	rs7119334	100	0.7376	0.284	A:C
	rs71311904	99.7			-:CATTT
	rs56164415	97.2	0.3048	0.025	G:A
HTR1A	rs6295	99.7	0.7971	0.246	G:C
	rs10042486	79.7	0.0004	0.249	T:C
	rs6449693	100	0.8162	0.188	A:G
	rs878567	100	1	0.19	G:A
	rs6294	99.3	1	0.054	C:T
HTR1B	rs6297	100	0.9752	0.126	T:C
	rs6296	100	0.5891	0.431	G:C
	rs6298	99.7	0.3177	0.427	G:A
S100A10	rs113674295	88.49			-: AGAAAAGCTCTGCTGTGTA

%gene: the percentage non-missing for this SNP site; HWpval: the Hardy Weinberg equilibrium p-value; MAF: the minor allele frequency for this SNP site; alleles: the major and minor alleles for this SNP site.

#### 3.2.3 Performance of different classifiers

To reveal the potential value of methylation level, clinic indicators and SNP features in the evaluation of DPs during 2 weeks follow-ups, a DL algorithm and six ML algorithms were constructed to implement the efficacy evaluation. The predictive performance of different classifiers is shown in Table 8. Ensemble learning algorithm combines multiple classifiers, which is better than a single classifier. GBDT classifier obtained the optimal AUC, which was 51.32 %. The optimal accuracy came from the random forest classifier, which was 58.86 %. High-dimensional data brings dimension disaster to supervised learning, which makes the prediction performance of the model unsatisfactory. Therefore, we adopt the feature selection method to select the relevant feature subsets from high-dimensional data in the follow-up.

**Table 8:** Performance of different classifiers on the dataset of methylation, SNP and clinic (All predictive performance is mean value).

	LR	DT	SVM	RF	GBDT	XGBC	DL
Accuracy	0.4928	0.5167	0.6171	0.5886	0.5593	0.5643	0.51757
Precision	0.5820	0.6096	0.6171	0.6122	0.6100	0.6139	0.436
Recall	0.6141	0.5891	1.0	0.9154	0.7904	0.7756	0.461
F1_score	0.5915	0.5942	0.7632	0.7329	0.6867	0.6824	0.41
AUC	0.4148	0.4946	0.4170	0.4741	0.5132	0.5085	0.425

#### 3.2.4 Feature selection

First, we used T-test to obtain a total of 14 differential features from methylation, clinical and SNP data sets, including 11 methylation features and 3 clinical indicators. At the same time, we screened 18 features from the methylation coding, clinical, and SNP datasets using T-test. There were 15 methylation features and 3 clinical features. Table 9 shows the prediction results of DL. A model was constructed to evaluate the prognostic effect of feature selection by T-test. Compared with the original data model, the performance of the model has been greatly improved, especially in terms of AUC and accuracy.

**Table 9:** Prediction results of DL algorithm of T-test.

dataset	Feature number	Accuracy	Precision	Recall	F1-score	AUC
Methylation + SNP +clinic	14	0.6690	0.63	0.602	0.55	0.722
Methylation encoding +SNP + clinic	**18**	**0.7076**	**0.736**	**0.67**	**0.644**	**0.742**

**Fig. 9:**
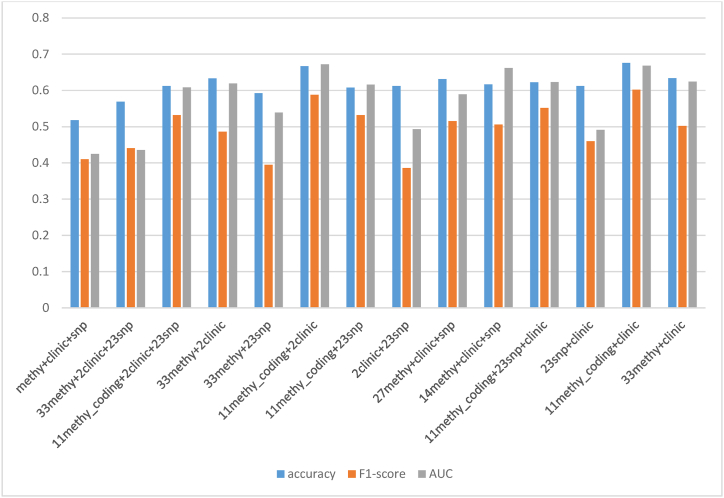
The prediction performance of the DL model of different feature subsets.

We then used RF to rank methylation features, methylation coding features, SNPs, and clinical markers according to feature importance scores. By sorting the feature importance scores of the total features of these data sets, the size of the optimal feature subset obtained is shown in Table 10. The optimal feature subset is used to build a DL model, and the model performance is evaluated by judging the indicators. RF feature selection algorithm improves the performance of the model (Fig. 9). Especially in the data set of 11 methylation coding features and clinical features, the accuracy rate increased by 30.54 %, F1_score increased by 46.83 %, and AUC increased by 57.18 %. Surprisingly, the prediction performance decreased when the dataset combined 2 clinical features with 11 methylation encoding features.

**Table 10:** The size of the optimal subsets of RF feature selection.

dataset	Feature number before dimensionality reduction	Feature number after dimensionality reduction
Methylation	406	33
Methylation encoding	406	11
SNP	68	23
clinic	27	2
Methylation + clinic + SNP	487	27
Methylation encoding + clinic + SNP	487	14

**Fig. 10:**
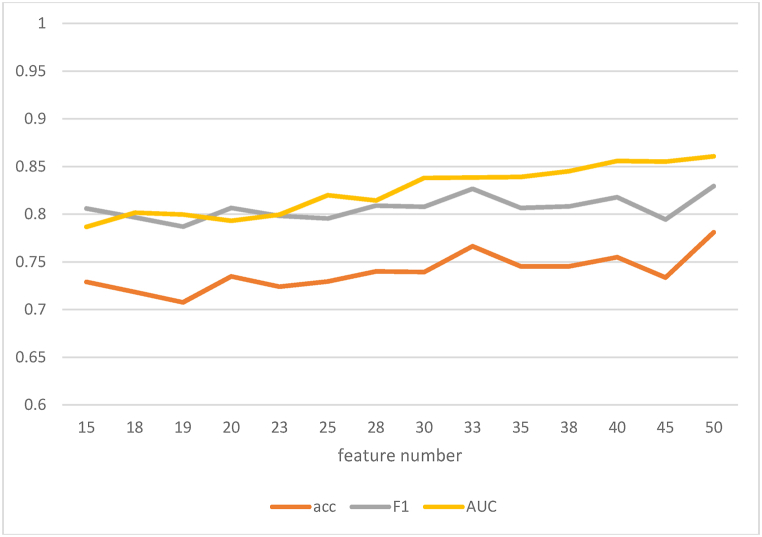
The relationship between the number of features and the evaluation indicators (accuracy, F1_score, AUC).

It is best that we use the RFE approach to characterize methylation, clinical, and SNP datasets to evaluate the efficacy of two-week DPs. The results show that there is a certain relationship between feature number and evaluation index (accuracy, F1_score, AUC). With the increase of features, the evaluation index will have a certain upward trend, as shown in Fig. 10. A sustained increase tends to be a smooth fluctuation. To balance the two purposes of reducing the number of features and improving the evaluation index of the model, 33 features were selected as the final feature subset. The prediction performance of 33 feature subsets is analyzed by DL algorithm, and the accuracy of the model is 75.94 %, the accuracy of F1_score is 73.99 %, and the AUC is 83.33 %. It has good prediction effect.

In general, we found that among the 33 features selected by the RFE method, the model had the best prediction effect, with an accuracy of 75.94 % and an AUC of 83.33 %, which could achieve an accurate evaluation of drug efficacy in patients with depression after 2 weeks.

## 4 Discussion

This study leveraged clinical data and sequencing data to elucidate notable disparities between individuals diagnosed with DPs and HCs, while also discerning significant variances between subgroups exhibiting effective versus ineffective prognostic outcomes within the DPs cohort. The viability of utilizing these discernible alterations as biomarkers for both diagnosing depression and predicting treatment efficacy was rigorously examined. Notably, our investigation represents the pioneering endeavor to amalgamate DNA methylation sequencing data, SNP data, and clinical parameters to construct predictive models distinguishing between MDD sufferers and healthy counterparts. Furthermore, we extend this innovative approach to forecasting antidepressant treatment outcomes among MDD patients through the application of DL methodologies.

Firstly, this study analyzed clinical data and sequencing data among depressed patients and healthy controls. Clinical factors in depression patients and healthy controls are evaluated in this study. The result found that NLES, CTQ and one-NLES scores were significantly different between depressed individuals and healthy controls. As shown in the results, higher scores of NLES, CTQ and one-NLES were found in depression patients compared with healthy controls. In conjunction with earlier studies, these results suggest that a combination of information from questionnaires and rating scales can meaningfully contribute to identifying depression patients. Meanwhile, this study provides a prediction model to differentiate between DPs and HCs using our method. We evaluated the performance of using clinic data, methylation data and SNP data alone or in combination in the identification model of depression patients. After a comprehensive validation and comparison, we demonstrated that multiple dimensionality features improved the prediction performance and feature interpretability of the prediction model using six ML models. The result further confirmed the ensemble learning models were greater than single prediction models [44]. In this study, we pinpointed that RF model outperformed other ML models in terms of AUC for distinguishing depression patients and healthy controls.

We focused on DL model, though six ML methods were compared. Our study achieved a high accuracy (82.383 %) between DPs and HCs with the dataset of methylation, clinic and SNP. Notably, it has a lower AUC (52.8 %) and F1_score (44.6 %). AUC is a better measure than accuracy based on formal definitions of discriminancy and consistency. The model showed high false positive considering that high-dimensional and low-sample datasets will affect the overall prediction performance of the model. To improve the performance of DL model, three different feature selection methods were used to identify the relevant feature subsets in the high-dimension data and find the optimal feature subset. Finally, we identified 18 predictive variables of identification DPs from RFE, including 15 methylation features, 2 clinical biomarkers and 1 SNP site. 2 clinical biomarkers encompass CTQ and one-NLES. Previous studies have shown that early life stress was associated with depression episodes [45]. The risk of depression will increase six-fold with six months of negative life events. Kessler conducted a study on negative life events of depression. The result showed that the more negative life events and the more serious they were, the higher the incidence of depression and the more serious the symptoms of depression would be. There was a relationship between the number of negative events and response [46]. In the present study, we evaluated the role of rs10042486 in DPs through genetic association analysis. The major finding was a significant association of rs10042486 within HTR1A promoter region with depression in the Han Chinese population. This finding might implicate the susceptibility of rs10042486 to depression. Surprisingly, this finding has been consistent with the rs10042486 was significantly associated with schizophrenia from Zhang R [47]. Moreover, of the 15 DNA methylation sites, seven CpG sites were significantly different between DPs and HCs, one at HTR1A, two at HTR1B, and four at the BDNF gene. Three of the four CpG sites on the BDNF gene were highly methylated. Studies have found that increased methylation of BDNF gene is associated with depression [48]. HTR1B is involved in serotonin synthesis, which affects behavior and anxiety in animal models [49,50]. The interaction of recent stressful life events showed a significant correlation between HTR1B SNP (rs6296C) and depression, but there was no significant difference in the number of life events, suggesting that the main contributor to this phenotype was epigenetic life events affecting the HTR1B gene [51]. In addition, HTR1A autoreceptors medicate feedback inhibition of 5-HT neurons, which was found to be associated with MDD [52]. There are studies finding that HTR1A SNP rs6295G allele mutations were associated with increased susceptibility to depression [51]. These results further suggest that the differential features of gene methylation can be used as potential biomarkers to guide the early diagnosis of depression.

After this, we combined the 15 methylation predictors with 2 clinical biomarkers and 1 SNP site to establish the predictive models for depression identification by using the DL model. Our result indicated that our DL model may provide a suitable approach to create predictive models for identification of depression patients with 93.4 % AUC, 89.16 % accuracy and 71.3 % F1_score. Exhilaratingly, there is an overlap between the subset determined by RFE and the subsets gained from RF and T-test. This further suggests that these features can be used as a valid indicator to explain the difference between DPs and HCs. The feature subset determined by RF obtained the best prediction accuracy (85.1 %). Our statistical learning approach allowed us to identify and rank the variables that contributed the variables that contributed the most and understand the mechanism underlying multivariate outcome prediction, which achieved 86.3 % AUC. The result further indicated that DPs and HCs could potentially be distinguished by the 18 predictive variables. The prediction was accurate enough to be clinically meaningful. The present results show that a combination of relatively few genetic and clinical feature variables can effectively identify DPs and HCs, which achieved the purpose of saving computing resources and improving performance.

In the second part, we focused on the efficacy prediction in patients with depression. In this study, we constructed the prediction model based on multiple features to predict 2 weeks of treatment response of antidepressants for DPs at baseline. Clinical and sequencing information were considered simultaneously. Serotonin reuptake inhibitors are the most commonly used antidepressant. In this study, 60.84 % DPs were using SSRI drugs and 39.16 % were patients of non-SSRI drugs. However, SSRI is not effective for all DPs. Approximately 38.14 % of patients did not significantly relieve after the first antidepressant treatment. Consistent with this result, previous studies indicated functional genetic variants in HTR1A and HTR1B may be involved in individual differences in SSRI treatment response [53]. Subsequently, associations of DPs with clinical parameters and the efficacy were investigated. The result demonstrated that gender, relapse times, and HAMD-0W were significantly different in DPs. Relapse times of DPs were dominantly characterized by once or twice. Our investigation showed that the fewer recurrences, the higher number of efficacy of patients. The current series of results suggests that episodes of MDD sensitize a patient to further episodes, easily leading to recurrent episodes of the disease. Thereby, it may incur a greater risk of treatment resistance and persistent depression episodes in pathophysiological process, suggesting more episodes will lead to a reduced response to antidepressants [54]. Our finding is consistent with the majority of previous findings in the literature that indicate the number of episodes of MDD has a substantial causal relationship with the efficacy of patients [55]. Hence, the number of depression episodes can be one of the best predictors of antidepressant efficiency [56]. On the other hand, sex differences appear as a possible factor in predicting antidepressant response. After two weeks of antidepressant treatment, female patients represented a significantly higher proportion of effectivity. Previous studies also demonstrated women as having a more favorable response to antidepressant drugs than men [57]. Based on previous studies of depressed patients, Higher scores of HAMD-0W were found in ineffective depression patients. HAMD score can reflect the severity of depression, the more severe the depression, the worse the treatment effect may be. Study comparing the methylated discrepancies of HTR1A, HTR1B, S100A10, BDNF between ineffective patients and effective patients. The result found 18 CpG sites were significantly different between the effective group and the ineffective group. Genetic studies have shown that HTR1A gene variation is associated with depression, especially a functional HTR1A SNP rs6295 in the promoter region was found to be associated with antidepressant pharmacogenetics in the different population [51]. HTR1B, as a significant candidate gene, is also thought to be involved in the pathogenesis of MDD and antidepressant treatment [58,59]. Therefore, the methylation information of candidate genes can provide beneficial information in depression efficacy assessment. Additionally, we evaluated the role of the SNP sites of HTR1A, HTR1B, S100A10, BDNF genes in depression patients through genetic association analysis. However, a significant association was not observed between SNP site and depression patients in our study. Interestingly, other studies have found that HTR1B SNP rs6298C shows a good response to SSRI antidepressants (citalopram). This may be the result of the number of patients taking citalopram was too small to show a significant relationship.

Subsequently, we assess the combined utility of pre-treatment clinical information and sequencing data, in predicting antidepressant response at week 2 of pharmacotherapy treatment using ML and DL methods. Pharmacodynamics is a complex effect result, affected by a variety of related factors. The results of general ML models and DL model were not ideal, which may have a better performance after feature selection. Meanwhile, in the aspect of integral prediction performance, the ensemble learning prediction models demonstrated significant improvement compared with single-level prediction models. To get better classification accuracy, we applied different feature selection strategies to get the optimal feature subset. Our statistical learning approach allowed us to identify and rank the variables that contributed the variables that contributed the most and understand the mechanism underlying multivariate outcome prediction. Despite the prediction was largely driven by specific symptom profiles. Because sex and HAMD-0W contributed to the prediction of the outcomes. Interestingly, the present analysis shows that the methylation encoding variables play an important role in the prediction, suggesting a complex interplay between methylation encoding information and other predictors in determining treatment outcomes, which adds to the interpretability of the result.

We applied an importance-ranked, forward-selection modeling approach of RF to search for the most predictive input variables from primary datasets of methylation, clinic and SNP. The experimental results clearly demonstrated that the feature subsets determined by random forest have a certain degree of improvement on the prediction effect and generalization performance of the model. The higher classification 67.57 % accuracy was achieved by clinic variables in combination with 11 methylation encoding variables using DL model. However, there is a certain gap between the prediction results and our target.

Afterward, the RFE is introduced to realize the screening of feature subsets. Surprisingly, our study has achieved satisfied classification efficacy for distinguishing effective depression patients from ineffective depression patients based on the feature subset determined by RFE models. The DL prediction model is valuable and parsimonious, based on only 33 variables. The result found that the accuracy of the model was 75.94 %, an increase of 46.72 %, 73.99 % of F1_score, an increase of 80.46 %, and the largest increase was AUC, with an increase of 96.07 % from 42.5 % to 83.33 %. There are 30 DNA methylation features, 2 clinical indicators (HAMD score and age of the first onset) and 1 SNP (rs7119334C). As several studies have reported, age is an important factor associated with depression efficacy. The increase in age will cause adverse reactions in the course of depression. Older patients tend to have a worse course of depression than younger or middle-aged patients. However, it was found an important clinical index affecting the efficacy of depression was the age of the first onset rather than the age in the study. The older the age of first onset, the better the treatment effect of depression was (the effective rate was 54.29 % in 70 patients under 30 years old, and 64.44 % in 90 patients over 50 years old). The rs7119334 is a genetic polymorphism site located on BDNF gene, which is related to the mechanism of antidepressant. Previous studies have found that BDNF Val66Met SNP, as well as other two BDNF SNPs, were linked to a reduced response to antidepressant treatment suggesting that these SNPs may impact treatment responses to antidepressants [60,61]. And the predictions are reproducible in non-overlapping validation dataset. Moreover, the present results show that a combination of relatively few genetic and clinical variables can predict whether an individual with depression may remission with a specific antidepressant. Additionally, these results demonstrate that a combination of genomic and clinical information has the potential to serve as a clinical decision-support tool that may help the clinician to select an antidepressant that an individual is more likely to benefit from. In this way, clinicians can pay attention to and adjust treatment according to the response results of different patients in the follow-up treatment.

In summary, DL algorithm, leveraging both clinical and sequencing data, exhibits commendable accuracy and offers an objective and precise diagnostic approach for discerning depression and predicting its pharmacodynamic responses. Moreover, the discerned differential features, once selected, hold promise as candidate biomarkers, furnishing valuable insights for the early detection and prognostic evaluation of depression.

## Data availability statement

The data related to this study are not stored in publicly available repositories and are private data. All the data are available upon request to the corresponding author.

## Ethics statement

This study was approved by the Zhongda Hospital ethics committee (reference number: 2016ZDSYLL100-P01), and performed in accordance with the Helsinki Declaration. For all participants, we obtained written informed consent after a complete description of the study.

## Funding

This work was supported by Open Project Programme of the Key Base for Standardized Training for General Physicians, Zhongda Hospital, 10.13039/501100012595Southeast University (No. 2022ZYJD15) and Jiangsu Provincial Medical Youth Talent (No. QNRC2016825); 10.13039/100014717National Natural Science Foundation of China (32270607).

## CRediT authorship contribution statement

**Yunyun Hu:** Writing – original draft, Software, Methodology, Data curation. **Jiang Chen:** Writing – original draft, Visualization, Investigation, Data curation. **Jian Li:** Writing – review & editing, Supervision, Conceptualization. **Zhi Xu:** Writing – review & editing, Validation, Supervision, Conceptualization.

## Declaration of competing interest

The authors declare that they have no known competing financial interests or personal relationships that could have appeared to influence the work reported in this paper.

## Figures and Tables

**Table A1:** Differential characteristics of depression recognition screened by T-test

Depression recognition dataset	T-test filtering features			
DNA methylation + SNP + clinical synthesis dataset	HTR1A_2_215	HTR1B_3_215	S100A10_4_95	BDNF_5_40
	HTR1B_3_53	HTR1B_4_88	BDNF_1_70	BDNF_5_45
	HTR1B_3_61	HTR1B_4_128	BDNF_1_146	BDNF_5_111
	HTR1B_3_135	S100A10_3_92	BDNF_4_59	BDNF_5_179
	BDNF_7_58	BDNF_7_86	BDNF_7_106	BDNF_9_94
	Life	LES	rs10042486_C/T	
DNA coding methylation + SNP + clinical synthesis dataset	HTR1A_2_196	HTR1B_3_131	S100A10_2_202	BDNF_7_58
	HTR1A_3_135	HTR1B_3_135	S100A10_2_204	BDNF_7_86
	HTR1A_3_139	HTR1B_4_41	S100A10_3_63	BDNF_7_106
	HTR1B_1_54	HTR1B_5_88	S100A10_3_92	BDNF_9_69
	HTR1B_1_194	HTR1B_5_158	BDNF_1_146	BDNF_9_94
	HTR1B_3_46	S100A10_1_96	BDNF_5_109	BDNF_9_100
	HTR1B_3_50	S100A10_2_169	BDNF_5_182	BDNF_9_104
	HTR1B_3_53	S100A10_2_192	BDNF_6_140	Life
	rs10042486_C/T			

**Table A2:** Differential features of depression recognition screened by RFE method

Depression recognition dataset	RFE filtering features			
DNA methylation + SNP + clinical synthesis dataset	HTR1A_2_196	HTR1B_4_128	BDNF_3_250	CTQ
	HTR1A_2_215	HTR1B_5_165	BDNF_4_59	Life
	HTR1B_2_125	HTR1B_5_240	BDNF_5_45	rs10042486_C/C
	HTR1B_3_135	S100A10_2_34	BDNF_7_58	BDNF_7_106
	HTR1B_3_147	S100A10_4_95		

**Table A3:** Differential features of depression recognition in RF screening

Depression recognition dataset	RF filtering features				
DNA methylation data set	HTR1B_2_232	BDNF_9_94	S100A10_2_216	BDNF_9_84	
DNA methylation coding dataset	S100A10_2_204	BDNF_7_58	BDNF_9_104	HTR1B_3_50	S100A10_2_169
	S100A10_3_122	BDNF_9_100	BDNF_1_131	HTR1B_3_46	BDNF_3_259
	HTR1A_4_186	BDNF_4_59	HTR1B_1_54	S100A10_3_92	BDNF_6_134
	HTR1B_4_41	S100A10_4_161	BDNF_5_98	BDNF_7_192	S100A10_3_152
	HTR1B_5_88	HTR1A_3_40	HTR1A_2_40	HTR1A_3_139	BDNF_5_182
	S100A10_3_63	BDNF_3_257	HTR1B_2_228	BDNF_6_140	HTR1A_3_142
SNP dataset	rs7119334_C/A				
DNA coding methylation + SNP + clinical synthesis dataset	LES	HTR1B_4_44	HTR1B_4_128	BDNF_4_27	BDNF_4_27
	Life	BDNF_3_111	HTR1B_5_165	HTR1B_5_88	HTR1B_3_53
	HTR1B_3_50	HTR1A_4_54	BDNF_3_257	BDNF_9_130	HTR1A_2_136
	S100A10_2_204	HTR1B_3_46	rs6296_G/G	BDNF_4_83	BDNF_4_136
	Age	BDNF_9_135	S100A10_1_148	HTR1B_4_156	BDNF_1_152
	CTQ	BDNF_1_70	S100A10_4_97	HTR1B_4_98	BDNF_9_100
	one-LES	S100A10_2_88	BDNF_9_94	BDNF_5_109	BDNF_9_229
	BDNF_6_140	S100A10_2_47	S100A10_3_182	rs10042486_C/T	BDNF_4_107
	HTR1B_3_131	S100A10_2_108	S100A10_1_198	HTR1B_1_201	HTR1B_2_232
	HTR1A_4_62	HTR1A_3_173	S100A10_2_192	S100A10_1_133	BDNF_7_123
	BDNF_7_58	BDNF_3_194	BDNF_6_118	rs6298_A/A	S100A10_3_106
	BDNF_4_87	HTR1A_4_158	S100A10_3_102	HTR1B_2_258	S100A10_2_169
DNA methylation + SNP + clinical synthesis dataset	HTR1B_1_65	BDNF_5_111	BDNF_9_143	S100A10_2_88	HTR1B_2_139
	HTR1B_5_238	BDNF_9_245	HTR1B_3_147	S100A10_2_216	BDNF_4_34
	BDNF_5_173	BDNF_7_58	HTR1B_4_128	HTR1B_2_78	BDNF_7_43
	BDNF_4_92	HTR1A_4_62	S100A10_2_112		

**Table A4:** Differential characteristics of pharmacodynamic prediction of depression screened by T-test

Pharmacodynamic prediction dataset for depression	T-test filtering features			
DNA methylation + SNP + clinical synthesis dataset	HTR1A_2_230	S100A10_2_86	BDNF_1_146	BDNF_7_165
	HTR1A_3_171	S100A10_2_127	BDNF_4_112	BDNF_9_166
	HTR1B_4_84	BDNF_1_119	BDNF_7_153	HAMD
	sex_Female	sex_Male		
DNA coding methylation + SNP + clinical synthesis dataset	HAMD	S100A10_2_34	BDNF_7_68	BDNF_9_166
	HTR1A_2_186	S100A10_2_108	BDNF_7_153	sex_Female
	HTR1A_2_230	S100A10_2_216	BDNF_9_46	sex_Male
	HTR1A_3_64	S100A10_3_131	BDNF_9_72	BDNF_9_118
	HTR1B_3_74	BDNF_1_119		

**Table A5:** RFE the differential characteristics of pharmacodynamic prediction of depression

Pharmacodynamic prediction dataset for depression	RFE filtering features			
RFE filtering features	HTR1A_3_135	HTR1B_4_84	S100A10_3_65	BDNF_9_72
	HTR1A_3_142	HTR1B_5_178	S100A10_3_102	BDNF_9_166
	HTR1A_3_171	HTR1B_5_260	S100A10_3_131	BDNF_9_171
	HTR1A_4_62	S100A10_1_37	S100A10_3_133	HAMD
	HTR1A_4_230	S100A10_1_58	S100A10_3_150	first_morbidity_age
	HTR1B_1_254	S100A10_1_71	S100A10_4_127	rs7119334_C/C
	HTR1B_2_232	S100A10_2_86	BDNF_1_119	BDNF_1_176
	HTR1B_3_111	S100A10_2_204	BDNF_7_153	BDNF_4_87
	BDNF_4_107			

**Table A6:** Differential characteristics of pharmacodynamic prediction of depression by RF screening

Pharmacodynamic prediction dataset for depression	RF filtering features			
DNA methylation dataset	BDNF_9_118	HTR1B_4_84	BDNF_7_179	BDNF_3_229
	BDNF_1_70	BDNF_9_166	BDNF_1_176	S100A10_3_106
	S100A10_3_131	S100A10_2_47	S100A10_4_115	S100A10_3_65
	S100A10_3_150	BDNF_9_72	HTR1A_2_186	BDNF_3_109
	HTR1B_1_254	BDNF_9_232	BDNF_7_123	S100A10_1_133
	HTR1B_2_90	BDNF_5_102	HTR1B_1_65	BDNF_9_250
	BDNF_4_87	BDNF_1_181	BDNF_7_47	HTR1B_3_37
	S100A10_1_156	S100A10_1_96	BDNF_9_61	HTR1B_3_61
	BDNF_3_26			
DNA coding methylation dataset	BDNF_9_118	S100A10_3_131	S100A10_1_96	BDNF_1_119
	BDNF_1_70	BDNF_9_166	S100A10_2_34	BDNF_7_153
	BDNF_7_68	HTR1B_2_90	BDNF_7_179	
Clinical dataset	age	SSRS score		
DNA coding methylation + SNP + clinical synthesis dataset	HAMD	SSRS points	BDNF_9_72	Relapse times
	first_morbidity_age	SHAPS score	BDNF_9_46	BDNF_1_119
	TAS score	BSI-CV score	HTR1B_3_74	BDNF_7_153
	age	BDNF_9_166		
DNA methylation + SNP + clinical synthesis dataset	BDNF_9_166	BDNF_3_154	BDNF_7_179	S100A10_1_133
	BDNF_1_119	BDNF_7_153	BDNF_3_194	HTR1B_1_254
	S100A10_2_86	BDNF_1_166	S100A10_2_127	BDNF_7_104
	BDNF_3_26	BDNF_9_46	HTR1B_2_90	BDNF_7_165
	HAMD-0W	HTR1B_3_74	HTR1B_3_147	HTR1A_3_40
	HTR1B_5_178	HTR1B_5_169	BDNF_9_118	HTR1A_1_122
	BDNF_9_72	S100A10_4_104	BDNF_4_83	

**Table A7:** Parameter summary

Model	Parameter
DL for depression prediction	optimizers = SGD; dropout = 0.4; lr = 0.02; epoch = 300; batch_size = 32 loss = binary_crossentropy
DL for prognostic efficacy assessment of depression	optimizers = SGD; dropout = 0.4; lr = 0.01; epoch = 200; batch_size = 32 loss = binary_crossentropy

**Table A8:** DL framework for depression prediction

Level	Type	Shape
Input Layer	Input Layer	(, 18)
Hidden Layer	Fully connected layer	(18, 4)
	Dropout Layer	
	Activation function (ReLU)	
Output Layer	Fully connected layer	(4, 1)
	Sigmoid Output Layer	

**Table A9:** DL for prognostic efficacy assessment of depression

Level	Type	Shape
Input Layer	Input Layer	(, 19)
Hidden Layer	Fully connected layer	(19, 12)
	Dropout Layer	
	Activation function (ReLU)	
	Fully connected layer	(12, 4)
	Dropout Layer	
	Activation function (ReLU)	
Output Layer	Fully connected layer	(4, 1)
	Sigmoid Output Layer	
